# Zero-gap semiconductor to excitonic insulator transition in Ta_2_NiSe_5_

**DOI:** 10.1038/ncomms14408

**Published:** 2017-02-16

**Authors:** Y. F. Lu, H. Kono, T. I. Larkin, A. W. Rost, T. Takayama, A. V. Boris, B. Keimer, H. Takagi

**Affiliations:** 1 Department of Physics, University of Tokyo, Bunkyo-ku, Tokyo 113-0033, Japan; 2 Max Planck Institute for Solid State Research, Heisenbergstrsse 1, 70569 Stuttgart, Germany; 3 Institute for Functional Matter and Quantum Technologies, University of Stuttgart, Pfaffenwaldring 57, 70550 Stuttgart, Germany

## Abstract

The excitonic insulator is a long conjectured correlated electron phase of narrow-gap semiconductors and semimetals, driven by weakly screened electron–hole interactions. Having been proposed more than 50 years ago, conclusive experimental evidence for its existence remains elusive. Ta_2_NiSe_5_ is a narrow-gap semiconductor with a small one-electron bandgap *E*_G_ of <50 meV. Below *T*_C_=326 K, a putative excitonic insulator is stabilized. Here we report an optical excitation gap *E*_op_ ∼0.16 eV below *T*_C_ comparable to the estimated exciton binding energy *E*_B_. Specific heat measurements show the entropy associated with the transition being consistent with a primarily electronic origin. To further explore this physics, we map the *T*_C_–*E*_G_ phase diagram tuning *E*_G_ via chemical and physical pressure. The dome-like behaviour around *E*_G_∼0 combined with our transport, thermodynamic and optical results are fully consistent with an excitonic insulator phase in Ta_2_NiSe_5_.

## Abstract (web summary)

The nature of an insulating phase in Ta_2_NiSe_5_ is an open question. Here, Lu *et al*. report transport, thermodynamic and optical evidences being fully consistent with an excitonic insulator phase in this material.

Formation of exotic electronic phases driven by electron correlations is among the most intriguing phenomena in condensed matter physics. One such many-body ground state is the excitonic insulator being stabilized in materials where the conduction and valence band are either separated by a small energy gap (semiconductors) or are overlapping in energy by a small amount (semimetals). In narrow-gap semiconductors and semimetals, the Coulomb interaction between electrons and holes may lead to a spontaneous formation of electron–hole pairs, namely, excitons^1^,^2^,^3^,^4^. These charge neutral pairs can in analogy to superconductivity give rise to an unconventional insulating ground state—the excitonic insulator—which is characterized by a many-body gap 2Δ_E_ opening in the single particle excitation spectrum (Fig. 1a). 2Δ_E_ mirrors the exciton binding energy *E*_B_.

The excitonic state is thermodynamically most stable for a zero-gap semiconductor *E*_G_=0. Reducing *E*_G_ to a negative value (the semimetallic region) increases the electron/hole density in the one-electron band structure due to band overlap. The increased carrier density screens the effective Coulomb interaction between electrons and holes, and consequently suppresses the stability of excitonic pairs and therefore *T*_C_. This is in strong contrast to a simple hybridization gap, which also exists for a large band overlap. For positive *E*_G_ (the semiconducting phase), the spontaneous formation of excitons is supressed with increasing *E*_G_ and the excitonic phase becomes unstable against the semiconducting ground state for *E*_G_*∼E*_B_ (refs 2, 4). In the semimetallic negative *E*_G_ region where interactions are well screened and weak, the transition may be described by BCS mean-field theory due to the analogy with superconductivity^2^,^4^,^5^,^6^. On going to the positive *E*_G_ region, one crosses over to a BEC-like limit as a consequence of strong coupling. This BCS–BEC crossover might be reflected in 2Δ_E_*/k*_B_*T*_C_ being strongly enhanced over the BCS weak coupling value. In Fig. 1a, we schematically show the generally predicted phase diagram with its characteristic dependence of the excitonic transition temperature *T*_C_ on the one electron bandgap *E*_G_ (refs 2, 4).

Candidate materials previously discussed include TmSe_0.45_Te_0.55_ (refs 7, 8, 9) and 1*T*-TiSe_2_ (refs 10, 11). In both cases, transitions to possible excitonic insulator phases were observed. These transitions are however accompanied by strong finite **q** lattice distortions as expected in the excitonic scenario for such indirect-gap semiconductors. Analysis of the entropy changes involved based on specific heat showed that the transitions in these materials are driven not only by the small number of electrons and holes but are strongly influenced by lattice and spin degrees of freedom^12^,^13^. This is in contrast to the excitonic insulator limit, where lattice deformations play a secondary role^1^,^2^,^3^,^4^,^5^,^6^,^14^. Alternative scenarios such as a charge density wave or a band Jahn–Teller effect, which were put forward especially for 1*T*-TiSe_2_ (refs 15, 16), can therefore not be excluded to describe the relevant physics of these materials, leaving room for question on the existence of a canonical excitonic insulator phase.

Ta_2_NiSe_5_, first reported by Sunshine *et al*.^17^ and Di Salvo *et al*.^18^, is a layered compound stacked by van der Waals interactions and crystallizing in an orthorhombic *Cmcm* structure above *T*_C_, as illustrated in Fig. 1b,c (ref. 18). Each layer is composed of parallel chains of edge-shared TaSe_6_ octahedra and corner-shared NiSe_4_ tetrahedra, running along the crystallographic *a* axis.

Band structure calculations in the orthorhombic high-temperature phase of Ta_2_NiSe_5_ show that the conduction band minimum and the valence band maximum are almost degenerate at the Γ point^14^,^19^ implying a very small direct gap *E*_G_. The valence band is composed of Ni 3*d* orbitals with a Se 4*p* admixture while Ta 5*d* orbitals dominate the conduction bands. Note that the valence and the conduction bands in the orthorhombic phase belong to different irreducible representations of the crystal structure^14^,^19^. The formation of a single particle hybridization gap is therefore forbidden by crystal symmetry.

The small direct gap semiconductor realised in Ta_2_NiSe_5_ above *T*_C_ is an ideal situation for the formation of an excitonic insulator phase. A semiconductor-to-insulator transition is indeed observed at *T*_C_≈326 K (ref. 18). This is accompanied by a very weak **q**=0 structural phase transition to monoclinic *C2/c* with the unit cell size remaining the same across the transition and whose small magnitude cannot account for the observed electronic changes^14^. This excludes a lattice distortion-mediated charge density wave mechanism driving the phase formation, as discussed in the case of Mo bronze^20^, suggesting it to be electronic in origin^14^. Recent X-ray photoemission spectroscopy (XPS) and angle-resolved photoemission spectroscopy (ARPES) measurements revealed a flattening of the valence band below *T*_C_, which was interpreted as the formation of an additional gap^21^. Based on these observations, the low-temperature phase was proposed to be an excitonic insulator. Ta_2_NiSe_5_ is distinct from other excitonic insulator candidates. First, due to the direct gap, the excitonic states do not require a finite **q** super-lattice modulation and can be accessed easily by optical spectroscopy. Second, electrons in Ta chains and holes in Ni chains are spatially separated in real space, which could stabilize the excitonic pair and hence the excitonic insulator.

Recognizing its uniqueness as a candidate excitonic insulator, electric transport, optical and specific heat measurements as well as physical and chemical pressure experiments were conducted on Ta_2_NiSe_5_ single crystals to test the validity of the excitonic insulator scenario. We will demonstrate that Ta_2_NiSe_5_ is a nearly zero-gap semiconductor at high temperatures with an optical gap *E*_op_*∼*0.16 eV, comparable to the expected excitonic binding energy *E*_B_, developing below *T*_C_=326 K. The majority of entropy change at the transition appears to originate from thermally excited electrons and holes consistent with the thermodynamics of phase formation to be dominated by electronic degrees of freedom. The physical and chemical pressure experiments indicate the suppression of *T*_C_ both by moving towards negative (semimetal) and positive (semiconductor) bandgap *E*_G_ resulting in a dome-shaped *T*_C_ versus *E*_G_ curve around *E*_G_=0. All these results are consistent with those expected for a canonical excitonic insulator.

## Results

### Zero-gap semimetal-to-semiconductor transition

**Figure 2:**
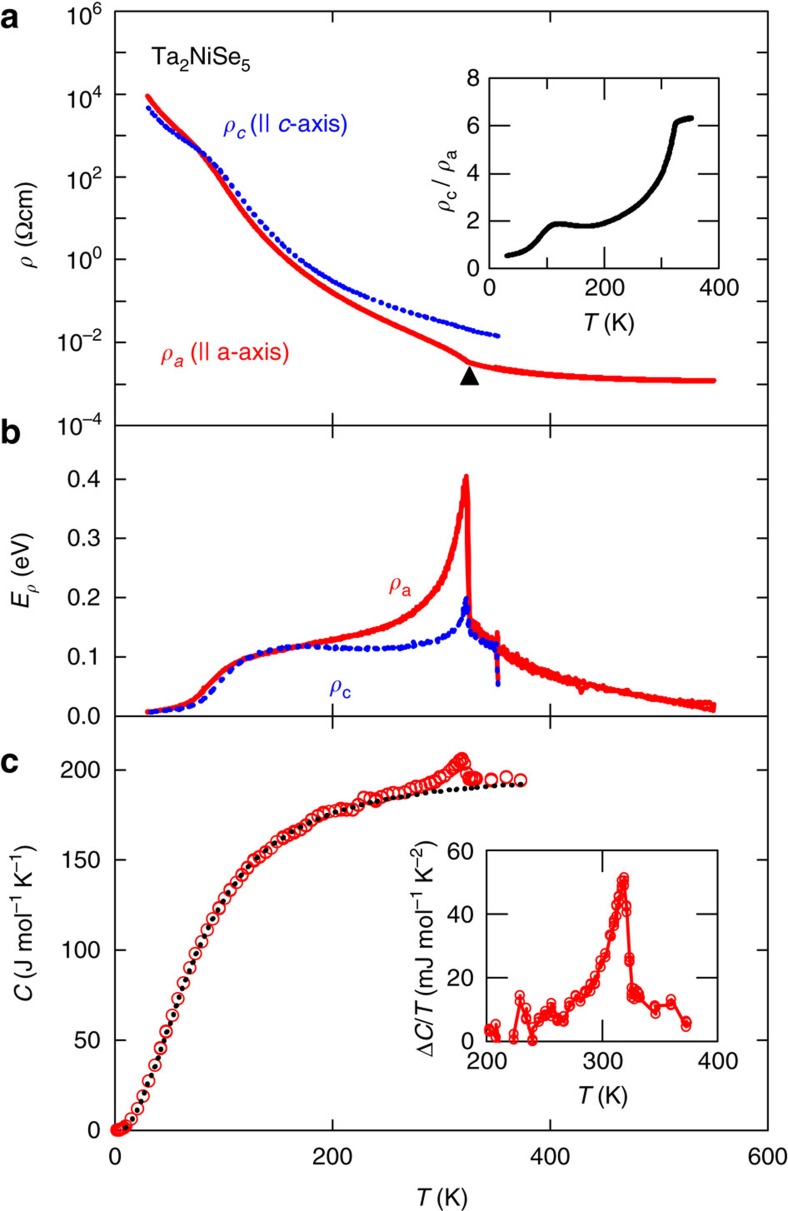
Ta_2_NiSe_5_ transport and specific heat. (**a**) Temperature dependence of the in-plane resistivity of Ta_2_NiSe_5_ single crystal *ρ*_a_ (red) along the *a* axis parallel to the Ta and Ni chains, and *ρ*_c_ (blue) along the *c* axis perpendicular to the chains. An anomaly is observed at *T*_C_=326 K (emphasized by black triangle). The inset shows the temperature dependence of the resistivity ratio, *ρ*_c_/*ρ*_a_ as an indicator of the in-plane anisotropy. (**b**) The temperature dependence of the activation energy *E*_*ρ*_ of the resistivity data in **a** given by *E*_*ρ*_=−*k*_B_*T*^2^(‖ln*ρ*/‖*T*). (**c**) Temperature dependence of heat capacity *C* of a Ta_2_NiSe_5_ single crystal. An anomaly in *C* at *T*_C_ is clearly visible. The phonon contribution (black dashed line) is roughly estimated from a Debye fit to the low-temperature data below *T*_C_. The inset shows the excess specific heat Δ*C*/*T* around *T*_C_ obtained from the raw heat capacity by subtracting the lattice contribution.

The transition in Ta_2_NiSe_5_ crystals is most readily seen in transport as shown in Fig. 2a. Here we present the resistivity *ρ*_a_ parallel to the Ta and Ni chains (*I* || *a* axis) as a function of temperature. A weak but well-defined kink is observed at *T*_C_=326 K. This agrees well with previous reports^18^. At high temperatures, the resistivity is essentially temperature independent over a wide range from 400 to 550 K. This means the transport gap is at most of the order of ∼0.05 eV, suggestive of an almost zero energy gap above *T*_C_. Right below *T*_C_, the resistivity increases rapidly, suggesting the opening of an additional gap. It is instructive for an excitonic insulator transition to present the data in form of an activation energy plot showing *E*_*ρ*_=−*k*_B_*T*^2^(‖ln*ρ*/‖*T*) as a function of temperature (Fig. 2b; Supplementary Fig. 1). *E*_*ρ*_ reflects an activation energy *E*_A_ if the temperature dependence at a given temperature is approximated by a thermally activated behaviour *ρ*=*ρ*_0_exp(−*E*_A_/*k*_B_*T*) and therefore mirrors the energy scale of thermal excitation of charge carriers. *E*_*ρ*_ shows a clear jump at *T*_C_ consistent with theoretical calculations^4^,^22^, predicting a step-like increase of *E*_*ρ*_ at *T*_C_. *E*_*ρ*_ below the jump is 0.1−0.2 eV, which suggests a gap of the order of a few tenths of an eV opening below *T*_C_. At lower temperature below 100 K, we observe a suppressed activation energy *E*_*ρ*_. In this temperature regime, the conduction due to impurity states, with much lower energy scale of charge excitations, very likely dominates the temperature dependence.

The resistivity perpendicular to the layer is orders of magnitude higher than the in-plane resistivity and we were not able to obtain a reliable estimate of the out-of-plane resistivity. Reflecting the presence of quasi-one-dimensional Ta and Ni chains, a moderate in-plane anisotropy is observed. As shown in Fig. 2a, above *T*_C_, the resistivity perpendicular to the chains (*I* || *c* axis), *ρ*_c_, is larger than *ρ*_a_ along the chains by a factor of 6, indicating weak but appreciable electronic quasi-one-dimensionality. Below *T*_C_, this quasi-one-dimensionality in transport appears to be washed out, suggesting that the excitonic gap formation makes charge transport isotropic. This isotropy might be related to the flattening of the one-dimensional dispersion. We note that such temperature dependence in the *ρ*_c_/*ρ*_a_ plot is absent in the family compound Ta_2_NiS_5_, which does not show any signature of an excitonic-like transition (Supplementary Fig. 2).

### Gap formation below *T*_C_

**Figure 3:**
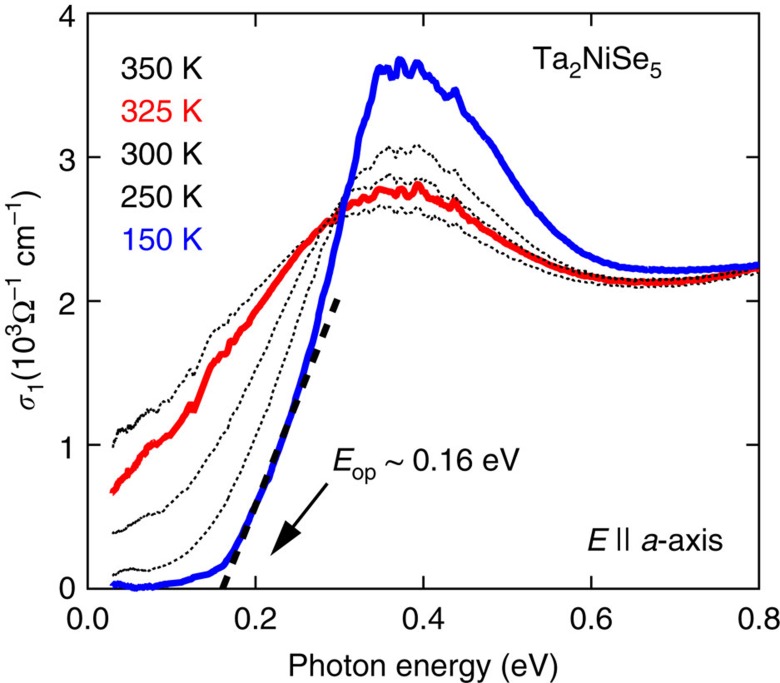
Optical conductivity. Optical conductivity spectra of Ta_2_NiSe_5_ single crystal at various temperatures, showing the opening of a charge excitation gap associated with the transition at *T*_C_=326 K. The gap of *E*_op_≈0.16 eV is estimated from the usual extrapolation of the linear regime of conductivity (black dashed line). The electric polarization was parallel to the *a* axis.

An additional many-body excitation gap 2Δ_E_ should open at *T*_C_ due to the excitonic transition. While ARPES^21^ measurements are consistent with such a gap opening, a complete picture of the gap formation has not been unveiled yet due to the limitation of the measurement to occupied states below the Fermi level and the restricted temperatures at which the experiments were carried out. The formation of an excitation gap at *T*_C_ is much more clearly evidenced by the optical conductivity spectrum with photon polarization along the chain direction *a* shown in Fig. 3. At high temperatures above *T*_C_, a broad continuum is observed with a hump-like structure around 0.3–0.4 eV, which is consistent with Ta_2_NiSe_5_ being an almost zero-gap semiconductor. On cooling below *T*_C_, the spectral weight is transferred from below an isosbestic point of E*∼0.3 eV to higher energies with a full gap of *E*_op_∼0.16 eV forming. At 150 K, a gap structure with an absorption peak at 0.4 eV is well established. The opening of a gap is further demonstrated by the temperature evolution of the conductivity at an energy well below the gap which we show in the Supplementary Fig. 3. According to the previous ARPES study, the band maximum relative to the Fermi energy—the effective valence band gap—was estimated to be 0.17 eV, which is roughly consistent with the characteristic energy for spectral weight transfer (isosbetic point) E*∼0.3 eV and the optical gap *E*_op_∼0.16 eV in the optical conductivity.

### Thermodynamic signature of phase transition

A clear anomaly in the specific heat *C*(*T*) can be identified at *T*_C_ (Fig. 2c), with the shape being reminiscent of a BCS-type superconducting transition. This constitutes thermodynamic evidence for the opening of a gap in the electronic excitation spectrum at this transition. The entropy associated with the transition is roughly Δ*S*∼0.3 J mol^−1^ K^−1^. The density of states *D* at the quasi-one-dimensional band edge is of the order of 1 state per eV per unit formula^14^, yielding a rough estimate of density of electrons and holes at *T*_C_=326 K of *D* × *k*_B_*T*_C_∼0.03 per unit formula in the zero-gap case. This is not inconsistent with the Hall coefficient +*R*_H_∼10^−2^ cm^3^ C^−1^ (hole like) measured at *T*_C_ (Supplementary Fig. 4), neglecting the ambiguity arising from the coexistence of electrons and holes. The quenched entropy of 3*k*_B_ for 0.03 carriers corresponds to an entropy change Δ*S∼*0.03 × 3 *R*=0.7 J mol^−1^ K^−1^, agreeing with the experimental value within a factor of 2. This rough estimate implies that the majority of entropy change associated with the transition and therefore the thermodynamically relevant degrees of freedom originate from the electronic entropy indicating an electronically driven phase transition.

### Phase diagram as a function of energy gap

The isovalent substitution of Se with Te and S was conducted to control the bandgap *E*_G_. Ta_2_NiS_5_ has compared with the isostructural Ta_2_NiSe_5_ a larger transport activation energy of *E*_*ρ*_∼0.2 eV in resistivity (inset, Fig. 5), indicative of the presence of a much larger bandgap *E*_G_ than in Ta_2_NiSe_5_. As discussed, band calculations indicate that the valence band of Ta_2_NiSe_5_ is composed of Ni 3*d* and Se 4*p* orbitals^14^. With increased S-substitution, the hybridization between Ni 3*d* and Se 4*p* (S 3*p*) orbitals are weakened. This lowers the energy of the states at the top of the valence band while the conduction band is less affected, resulting in an overall enhancement of the bandgap *E*_G_ compared to Ta_2_NiSe_5_. Changes in the Ta-chalcogen hybridization further enhance the band gap with increasing S-substitution but are predominantly affecting the energetically higher lying Ta-*d* bands above 1 eV (ref. 23). Tellurium doping, on the other hand, pushes the system in the opposite direction, likely bringing the nearly zero gap of Ta_2_NiSe_5_ to a negative region. Single crystals of the isovalent series Ta_2_Ni(Se_1−*x*_S_*x*_)_5_ and Ta_2_Ni(Se_1−*x*_Te_*x*_)_5_ were grown. Te doping was limited up to *x*=0.2 due to the solubility limit likely due to the much larger ionic radius of Te.

**Figure 4:**
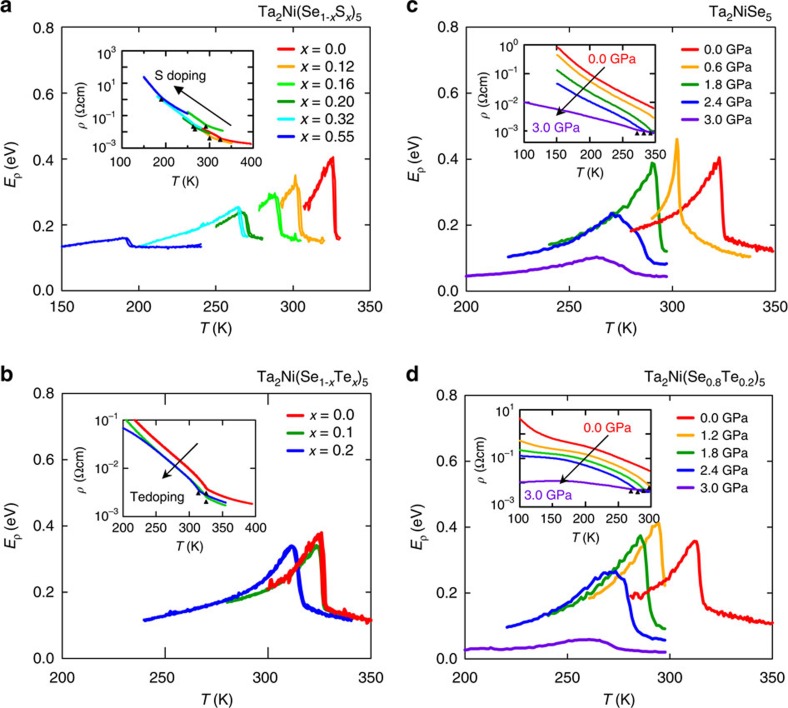
Chemical and physical pressure. (**a**) The activation energy plot *E*_*ρ*_=−*k*_B_*T*^2^(‖ln*ρ*/‖*T*) for Ta_2_Ni(Se_1−*x*_S_*x*_)_5_ that clearly shows the systematic shift of *T*_C_ with S doping. The inset shows resistivity curves (*I* || *a* axis) as a function of temperature. Triangles mark the transition temperatures for each sample. Variations in absolute values are due to uncertainties in, for example, exact sample dimensions. In **b**, we show the equivalent data for the Ta_2_Ni(Se_1−*x*_Te_*x*_)_5_ series. (**c**) The activation energy plot as a function of temperature for a single high-pressure experiment showing both the systematic reduction of *E*_*ρ*_ with pressure as well as the suppression of *T*_C_. Additional data from a high-temperature run are also included. The inset shows the equivalent resistivity data with emphasis of *T*_C_ by triangles. (**d**) Equivalent data to **c** for Ta_2_Ni(Se_0.8_Te_0.2_)_5_.

**Figure 5:**
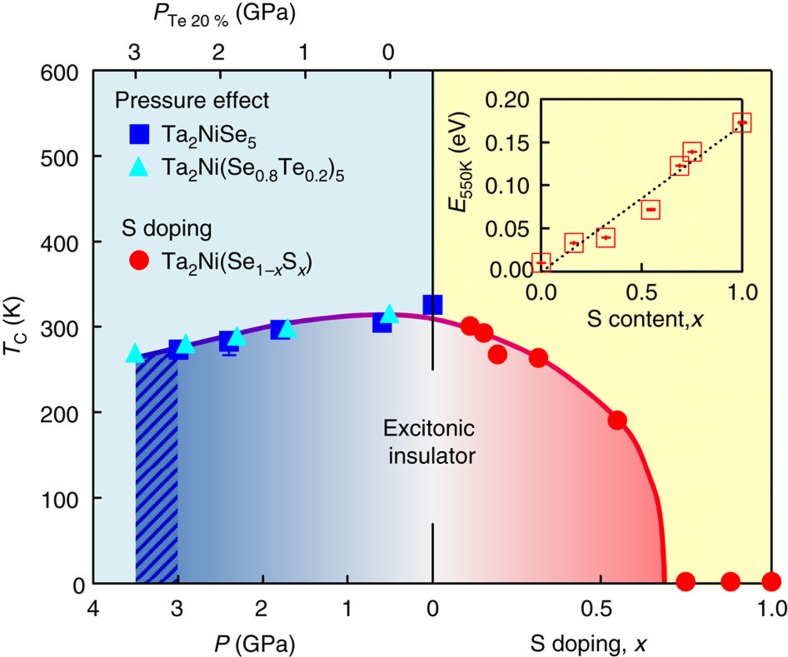
Phase diagram. Phase diagram of the excitonic insulator phase in Ta_2_NiSe_5_ given by the transition temperature *T*_C_ as a function of S doping (right) and pressure and Te doping (left), corresponding to the semiconducting and the semimetallic regions in the predicted phase diagram respectively. The pressure effect on Ta_2_Ni(Se_0.8_Te_0.2_)_5_ is also shown in the left region with an offset of chemical pressure of 0.5 GPa (see text). The shaded area in the left region indicates the region for phase separation behaviour at high pressures (see text), likely originating from a first-order transition. The inset shows the S doping *x* dependence of the transport activation energy of resistivity at high temperatures (500–550 K), *E*_550 K_. *E*_550 K_ gives a measure of the one electron gap *E*_G_ and indicates that *E*_G_ increases monotonically as a function of *x* from almost zero for *x*=0 to at least a few tenths of an eV for *x*=1.0. The sulfur substitution level *x* and pressure *P* may therefore be mapped onto the single band gap *E*_G_ in the high-temperature phase.

We show the activation energy plot of resistivity for Ta_2_Ni(Se_1−*x*_S_*x*_)_5_ and Ta_2_Ni(Se_1−*x*_Te_*x*_)_5_ in Fig. 4a,b, respectively. It is clear that both by increasing and by decreasing the band gap *T*_C_ is suppressed, meaning *T*_C_ is peaked for Ta_2_NiSe_5_ with a nearly zero bandgap. The inset of Fig. 5 shows the evolution of the activation energy *E*_550K_ for Ta_2_Ni(Se_1−*x*_S_*x*_)_5_ estimated from high-temperature data in the temperature range of 500–550 K well above *T*_C_ (Supplementary Fig. 1), which should be closely related to the bandgap *E*_G_. It is monotonically increasing and can be described by a linear correlation to leading order as shown, implying the continuous and almost linear increase of the bandgap *E*_G_ upon S-substitution. As *E*_550K_ is systematically enhanced with increasing S-substitution, *T*_C_ is suppressed to lower temperatures and eventually, for *x*>0.55, no phase transition was observed in the temperature dependent resistivity down to 2 K. For Ta_2_Ni(Se_1−*x*_Te_*x*_)_5_, the decrease of resistivity at 350 K by ∼70% for *x*=0.1 and 0.2, respectively, indicates the continuous suppression of the energy gap (Fig. 4b). Although the range of substitution is limited, the plot of *E*_*ρ*_ indicates a small but well resolved decrease of *T*_C_.

To confirm the suppression of *T*_C_ by decreasing *E*_G_ towards the negative side, namely by Te-substitution, physical pressure was applied to Ta_2_NiSe_5_. As can be seen from the activation energy plot in Fig. 4c, a clear decrease of *T*_C_ is observed with increasing pressure. At 2.5 GPa, *T*_C_ is decreased from 326 to 280 K, corresponding to a *T*_C_ suppression rate of ∼20 K GPa^−1^, which can be reasonably ascribed to the decrease of the energy gap in the negative gap region. With 20% of Te-substitution, *T*_C_ is reduced by 10 K, which is equivalent to 0.5 GPa of pressure. We performed the same pressure experiment also on 20% substituted Ta_2_Ni(Se_0.8_Te_0.2_)_5_ and observed again the suppression of *T*_C_ with a rate of ∼20 K per GPa (Fig. 4d). Therefore, once we offset an effective chemical pressure equivalent to 0.5 GPa, the two *T*_C_ (*P*) curves for pure and 20% Te substitute crystals fall onto a universal curve, strongly suggesting that the phase transition is controlled by a single parameter *E*_G_. In the pressure range above 2.4 GPa, we observe that upon increasing pressure further the transition temperature does not shift significantly, while the magnitude of the anomaly is substantially supressed (Fig. 4c,d) and conductivity in the low-temperature limit is significantly enhanced, suggestive of a first-order phase transition and the resultant phase coexistence. A recent pressure study up to 10 GPa on Ta_2_NiSe_5_ indeed indicates that there is a first-order structural phase transition at *P*_C_∼2.5 GPa, and the system becomes metallic above *P*_C_^24^. A reduction of resistivity under pressure is also measured for Ta_2_NiS_5_ but no phase transition was observed up to 3 GPa.

The control of *T*_C_ by changing *E*_G_ via chemical substitution and physical pressure effects can be summarized in the phase diagram shown in Fig. 5, where the pressure is a measure of decreasing *E*_G_ on the negative *E*_G_ side and the S-substitution is the measure of increasing *E*_G_ on the positive side. Te-substitution was converted to equivalent pressure on the pressure axis. We clearly see the expected dome-shaped dependence of *T*_C_ around *E*_G_∼0 (Ta_2_NiSe_5_).

## Discussion

The high-temperature phase of Ta_2_NiSe_5_ appears to be very close to a zero-gap semiconductor making it an ideal candidate system for the stabilization of an excitonic insulator phase. Our optical data indeed show that below the phase transition temperature of *T*_C_=326 K, a gap *E*_op_∼0.16 eV develops in the excitation spectrum consistent with the enhanced activation energy observed in our transport data. Significant spectral weight shifts occur from below the isosbestic point of ∼0.3 eV to higher energy scales of up to ∼0.6 eV. These energy scales should reflect the characteristic energy scales of the excitonic insulator and in particular experimentally constrains the scale of the exciton binding energy *E*_B_ to be of the order of a few tenths of an eV. Expressed in terms of an effective temperature (0.1 eV corresponding to ≈1,000 K) *E*_B_ is significantly larger than the experimental transition temperature to the excitonic insulator phase at 326 K, enabling the formation of this phase in Ta_2_NiSe_5_.

The exciton binding energy *E*_B_ of the order of a few tenths of an eV is orders of magnitude larger as compared with those of conventional three-dimensional semiconductors. However, we argue that the unusual properties of Ta_2_NiSe_5_ explain at least in part the enhanced *E*_B_. The excitonic binding energies can be captured by *E*_B_=−13.6 eV*μ*/*m*_0_*ɛ*^2^. In this Rydberg formula, *μ* is a material specific effective mass and *ɛ* an effective permittivity^25^. First of all the *d*-band character of conduction (Ta 5*d*) and valence bands (Ni 3*d*) result in an effective reduced mass *μ* of the order of *m*_0_ as can be determined from ARPES data^21^ and band structure calculations^14^. This reduced mass is up to two orders of magnitude larger than in conventional direct gap semiconductors^26^. Furthermore the effective low dimensionality of the band structure and resulting physical properties are known to generally lead to a strong increase of the exciton binding energy^26^. Taken together, these effects can be expected to lead to a large enhancement of the exciton binding energy to a value consistent with tenths of an eV.

We now turn to the evolution of these properties under pressure and chemical substitution. The emerging phase diagram based on our transport measurements of *T*_C_ as a function of the bandgap *E*_G_ in the high-temperature phase shown in Fig. 5 is consistent with the theoretical expectation for the *T*_C_*–E*_G_ dependence^4^, further supporting the excitonic state. *T*_C_ is optimized for the nearly zero-gap semiconductor (*E*_G_∼0) Ta_2_NiSe_5_. On increasing *E*_G_ in the solid solution Ta_2_Ni(Se_1−*x*_S_*x*_)_5_, the excitonic state should be suppressed and switch into a normal semiconductor when *E*_G_∼*E*_B_. For Ta_2_NiS_5_ located outside the dome in the phase diagram, the high-temperature (500–550 K) transport activation energy of ∼0.2 eV, shown in the inset in Fig. 5, implies an energy gap *E*_G_ of ∼0.4 eV. Recent optical measurements show a similar trend with a gap of ∼0.25 eV at 350 K measured in Ta_2_NiS_5_ (ref. 23) compared with the effective zero gap in Ta_2_NiSe_5_ (Fig. 3). These results imply that, in contrast to Ta_2_NiSe_5_, *E*_G_ in Ta_2_NiS_5_ is larger than *E*_B_, which has a weaker dependence upon sulfur substitution^23^. These findings are fully consistent with the absence of an excitonic transition in Ta_2_NiS_5_. In between these limits around *x*=0.5, the energy scale of the bandgap *E*_G_ in the high-temperature phase can therefore be expected to become comparable with the exciton binding energy *E*_B_ when crossing over from several hundreds of meV in Ta_2_NiS_5_ to effectively zero in Ta_2_NiSe_5_. This accounts for the stabilization of the excitonic insulator phase around that doping level.

We now turn our attention to the left hand part of the phase diagram. With going to the negative *E*_G_ side with pressure, *T*_C_ goes down with decreasing *E*_G_. This is consistent with the suppression of the excitonic binding due to the increased screening. The system is approaching the weak coupling BCS regime within the excitonic insulator scenario. Consequently the near-zero-gap Ta_2_NiSe_5_ with the highest *T*_C_ is in the BCS–BEC crossover regime and a relatively strong coupling is anticipated. Indeed, the experimentally obtained *E*_op_/*k*_B_*T*_C_∼6.5, a measure of coupling strength, for Ta_2_NiSe_5_ is much larger than 3.52 for weak coupling BCS, pointing to a moderately strong coupling.

Finally, we are not aware of any other electronically driven mechanism that could give rise to the observed behaviour. Since there is no evidence for magnetic-order scenarios such as Slater- or Mott-insulating phases can be readily excluded. Neither is there any Fermi surface nesting possible that could give rise to a partial gapping of the band structure. Indeed, the excitonic insulator scenario is the only theoretical proposal we are aware of that is consistent with all the observed experimental facts. In particular, the theoretically conjectured dome-shaped phase diagram we observe in this system is the cleanest among all excitonic insulator candidates and therefore represents a key advance in the search for this phase.

In summary, due to the relatively large effective mass of electrons and holes, and the reduced effective dimensionality, the layered direct gap semimetal Ta_2_NiSe_5_ can have a large exciton binding energy of a few tenths of an eV. The separation of electrons and holes confined within the quasi-one-dimensional chains may further help stabilizing the formation of excitons. The optical, transport and specific heat measurements on Ta_2_NiSe_5_ single crystals indicate that the system has an almost zero energy gap at high temperature but, below an electronically driven transition at *T*_C_=326 K, an excitation gap of *E*_op_∼0.16 eV develops. The excitation gap is comparable to the expected exciton binding energy and is reasonably ascribed to the excitonic gap. The electronic phase diagram, *T*_C_ as a function of the energy gap shows a dome-shaped behaviour centred at *E*_G_=0 expected for an excitonic insulator. Furthermore, the excitonic phase indeed disappears on increasing the energy gap *E*_G_ when *E*_G_ becomes comparable to the exciton binding energy, as expected. Based on this analysis, we conclude that an excitonic state is realized below *T*_C_=326 K in the nearly zero-gap semiconductor Ta_2_NiSe_5_.

## Methods

### Sample preparation

Single crystals of Ta_2_NiCh_5_ (Ch=S, Se and Te) were synthesized by chemical vapour transport. Elemental powders of tantalum, nickel and chalcogens (S, Se and Te) were mixed with a stoichiometric ratio and sealed into an evacuated quartz tube (∼1 × 10^−3^ Pa) with a small amount of I_2_ as transport agent. The mixture was sintered under a temperature gradient of 950/850 °C. After sintering for 1 week, needle-like single crystals were grown at the cold end of the tube.

### Physical properties measurements

Electric resistivity and heat capacity measurements were carried out by using a PPMS (Quantum Design). Pressure experiments were carried out using a HPC-33 pressure cell by ElectroLab. High-temperature electronic resistivity was measured by using a bespoke measurement device. Optical parameter were determined using spectroscopic ellipsometry^23^.

### Data availability

All relevant data are available from the corresponding authors upon request.

## Additional information

**How to cite this article:** Lu, Y. F. *et al*. Zero-gap semiconductor to excitonic insulator transition in Ta_2_NiSe_5_. *Nat. Commun.* **8,** 14408 doi: 10.1038/ncomms14408 (2017).

**Publisher's note:** Springer Nature remains neutral with regard to jurisdictional claims in published maps and institutional affiliations.

## Supplementary Material

Supplementary Information Supplementary Figures 1-5

## Figures and Tables

**Figure 1:**
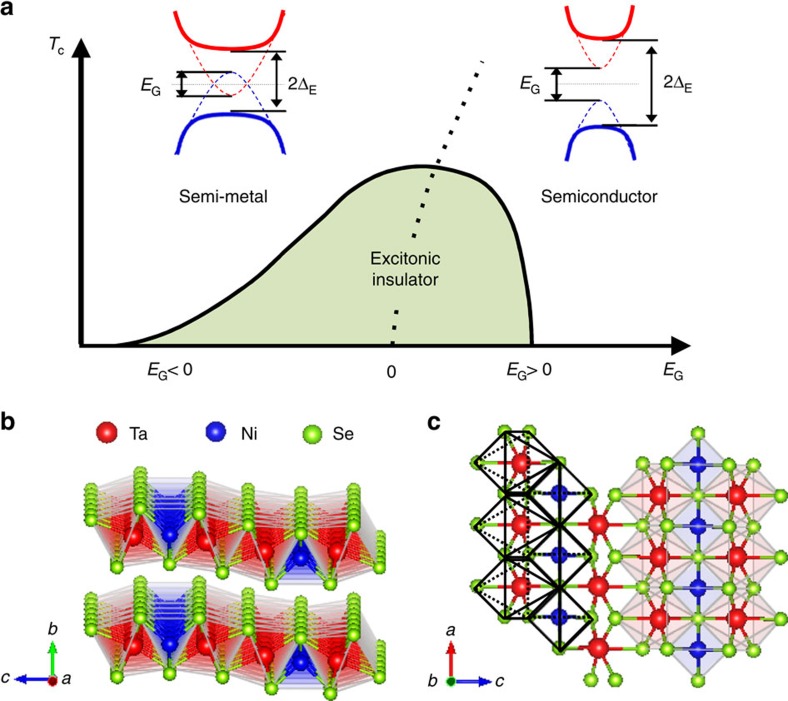
Excitonic insulator and Ta_2_NiSe_5_ structure. (**a**) Schematic phase diagram of excitonic gap formation as a function of *E*_G_. The dotted line indicates the crossover from semimetallic to semiconducting behaviour. The insets show the relation between *E*_G_ and Δ_E_ for the metallic and insulating limits. *E*_G_ is the one-electron bandgap between conduction (red) and valence band (blue). The Coulomb interaction between electron- and hole-like excitations can lead to electron–hole pairing (exciton formation) and the opening of a new many-body excitation gap Δ_E_ in the excitonic insulator phase. (**b**) The orthorhombic phase of Ta_2_NiSe_5_ shown in two orientations emphasizing the layered nature in the *a*–*c* plane, and (**c**) the TaSe_6_ and NiSe_4_ chains along the *a* direction (right).
