# Supplementary material for: Zero-gap semiconductor to excitonic insulator transition in Ta2NiSe5
Source: Nat Commun. 2017 Feb 16;8:14408. doi: 10.1038/ncomms14408 (PMC5316885; doi:10.1038/ncomms14408)
Supplement: Supplementary Information — Supplementary Figures 1-5 [file ncomms14408-s1.pdf]

Supplementary Figures

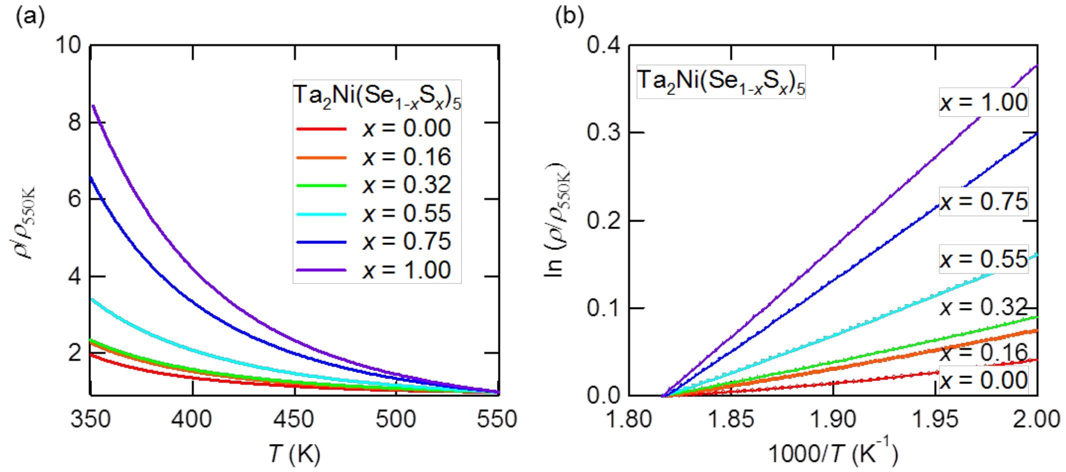

Supplementary Figure 1 | High temperature resistivity of sulfur substituted Ta_2NiSe_5 (a) Electronic resistivity of sulfur doped Ta_2NiSe_5 in the temperature range of 350 K to 550 K (normalised to 550 K). (b) Corresponding Arrhenius plot. The reported band calculations indicate that the valence band of Ta_2NiSe_5 is composed of Ni 3*d* and Se 4*p* orbitals. With increased sulfur doping, the hybridization between Ni 3*d* and Se 4*p* (S 3*p*) orbitals is weakened since the energy levels of the S 3*p* orbitals are lower than those of the Se 4*p* orbitals. This eventually lowers the energy level of the valence band while the conduction band is less affected, resulting in an overall enhancement of the energy gap compared to Ta_2NiSe_5. Tellurium doping, on the other hand, pushes the system in the opposite direction. The stronger hybridization between Ni 3*d* and Te 5*p* orbitals leads to the valence bands being at higher energy which drives the system to be more metallic. Supplementary Fig. 1a shows high temperature resistivity ($I \parallel a$-axis) in the temperature range of 350 K to 550 K. The corresponding Arrhenius plot of lnρ is shown in panel b. In the case of a simple semiconductor with energy gap E_G the resistivity ρ as a function of temperature T in the high temperature intrinsic regime is proportional to $\rho \propto \exp(E_G/2k_BT)$. The experimentally defined activation energy $E_\rho = -k_BT^2(\partial \ln\rho/\partial T)$ at high temperatures therefore reflects E_G. It is clearly seen that the experimental activation energy is systematically enhanced (larger slope) with increased sulfur content, x.

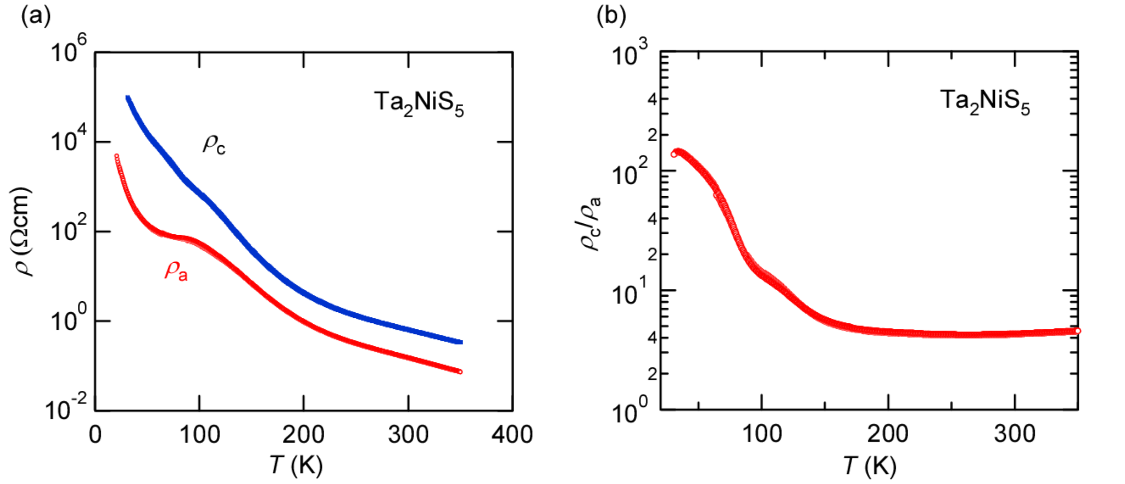

Supplementary Figure 2 | In-plane anisotropy of Ta_2NiS_5 (a) The electronic resistivity of Ta_2NiS_5. The electric current is applied along the *a*-axis (red) and *c*-axis (blue) (b) The electronic anisotropy ratio ρ_c/ρ_a. The quasi-one-dimensionality becomes significant with lowering temperature. In contrast to Ta_2NiSe_5, as discussed in the main text, Ta_2NiS_5 does not exhibit any phase transition down to 2 K. The in-plane anisotropy ratio (ρ_c/ρ_a) is estimated to be ~5 at 350 K, comparable to Ta_2NiSe_5. On cooling, ρ_c/ρ_a starts increasing and reaches ~100 at 50 K.

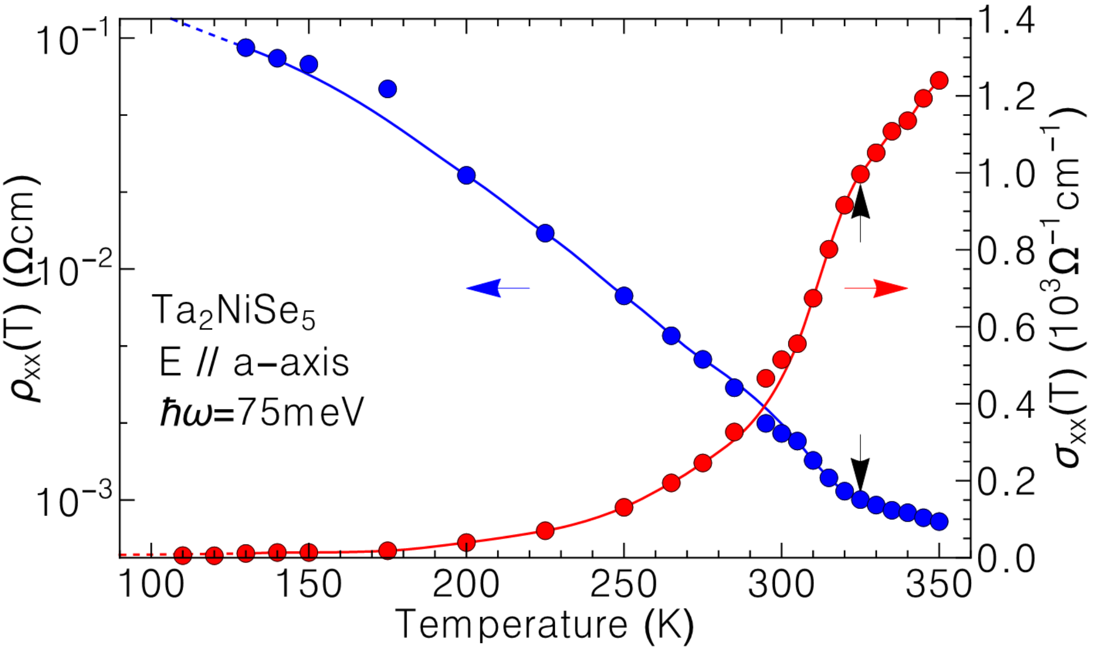

Supplementary Figure 3 | Temperature dependence of the infrared conductivity Infrared conductivity σ_{xx} and corresponding resistivity ρ_{xx}=1/σ_{xx} of Ta_2NiSe_5 vs. temperature measured at photon energy of 75 meV and electric polarization parallel to the *a*-axis. The filling of the electronic states within the optical gap ($\hbar\omega$ = 75 meV < $E_{op} \approx$ 160 meV), expressed in terms of the inverse optical conductivity (ρ_{xx} = 1/σ_{xx}) closely follows the behavior of the dc transport data (Fig. 2a) with a similar kink near the transition at T_C = 326 K.

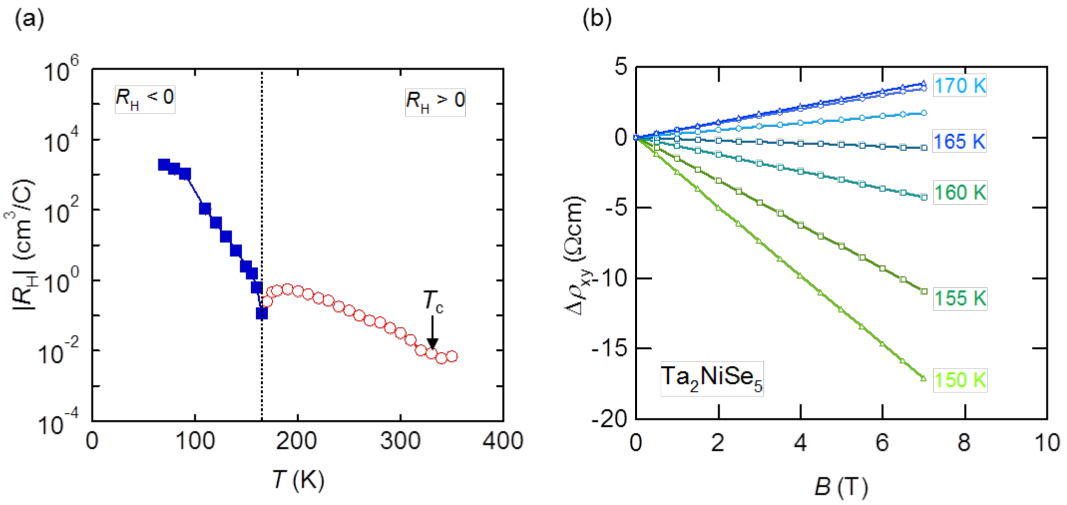

Supplementary Figure 4 | Hall signatures of Ta_2NiSe_5 (a) The measured Hall coefficient of Ta_2NiSe_5 in the temperature range of 70 K < T < 350 K and (b) Hall signal as a function of magnetic field. The black arrow indicates T_C. The R_H value was estimated to be +7 × 10^{-3} cm^3/C at 350 K and found to be increasing rapidly below T_C. The R_H changes its sign at ~170 K, evidencing the co-existence of electrons and holes.

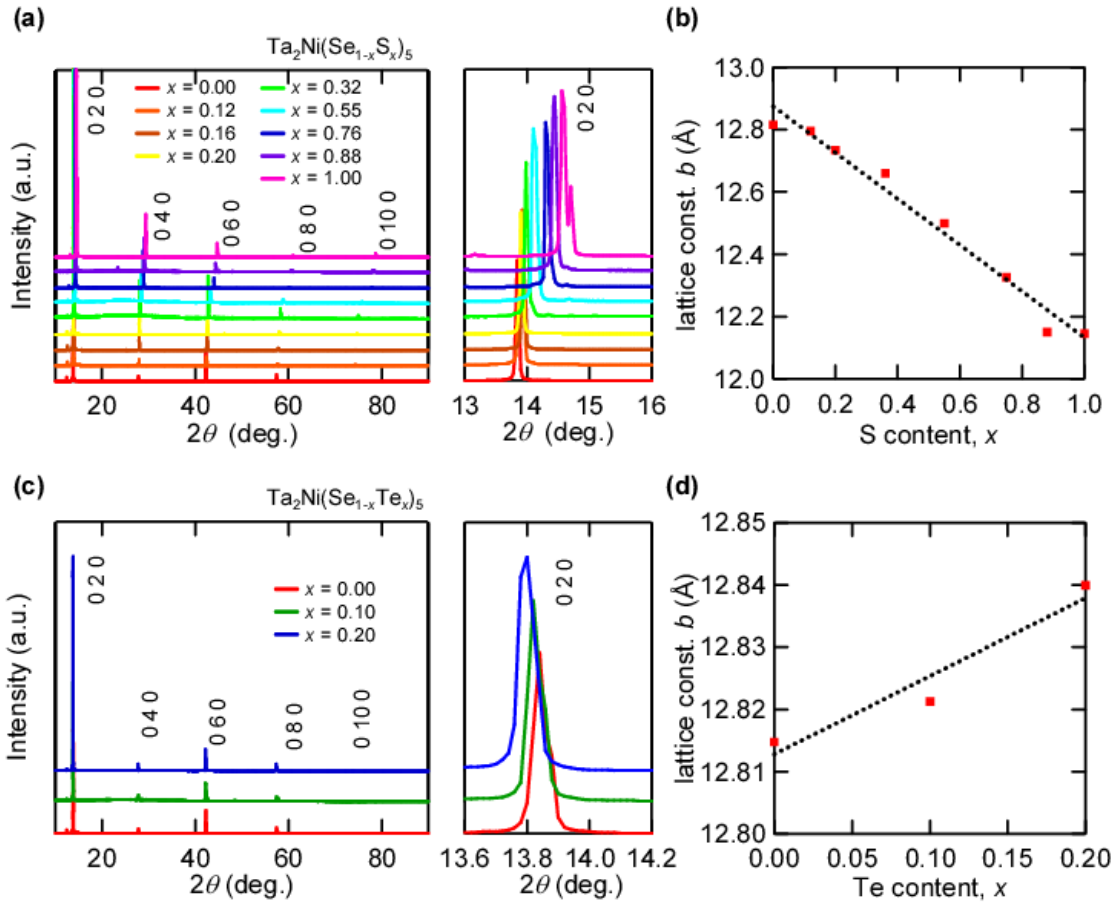

Supplementary Figure 5 | XRD analysis of pure and sulfur/tellurium-substituted Ta_2NiSe_5

(a) X-ray diffraction (XRD) pattern of sulfur- and (c) tellurium-substituted Ta_2NiSe_5 single crystals taken at room temperature. The (0 *b* 0) (*b* = 2, 4, 6, 8 and 10) peaks are systematically shifted towards higher and lower 2θ angle, respectively. The lattice constant *b* of the samples is summarized in (b) and (d) respectively. Single crystalline samples were synthesized using the chemical vapour transport method. Powders of elemental tantalum, palladium and sulfur were mixed with a starting composition of 2:1:5, and loaded into an evacuated quartz tube (~1×10^{-3} Pa) with iodine as transport agent. The mixture was sintered under a temperature gradient of 950/850 ^{o}C. After sintering for 1 week, needle-like single crystals were grown at the cold end of the tube. The lattice constant of $Ta_2Ni(Se_{1-x}S_x)_5$ shrinks as the sulfur content increases while tellurium substitution enlarges the lattice constant. It is concluded that chemically substituted Ta_2NiSe_5 was successfully synthesized since the change of lattice constant follows Vegard's rule.
